# Human Adenovirus Type 26 Infection Mediated by αvβ3 Integrin Is Caveolin-1-Dependent

**DOI:** 10.1128/spectrum.01097-22

**Published:** 2022-08-04

**Authors:** Davor Nestić, Jerome Custers, Danijel Švec, Dragomira Majhen

**Affiliations:** a Division of Molecular Biology, Ruđer Bošković Institute, Zagreb, Croatia; b Viral Vaccine Discovery and Early Development, Janssen Vaccines and Prevention BV, Leiden, The Netherlands; Instituto de Histología y Embriología de Mendoza (IHEM)

**Keywords:** αvβ3 integrin, human adenovirus type 26 infection, adenovirus cell entry, caveolin-1, clathrin, dynamin-2, internalization, non-enveloped DNA virus, vaccine vector, virus endocytosis

## Abstract

Human adenovirus type 26 (HAdV26) has been recognized as a promising platform for vaccine vector development, and very recently vaccine against COVID-19 based on HAdV26 was authorized for emergency use. Nevertheless, basic biology of this virus, namely, pathway which HAdV26 uses to enter the cell, is still insufficiently known. We have shown here that HAdV26 infection of human epithelial cells expressing low amount of αvβ3 integrin involves clathrin and is caveolin-1-independent, while HAdV26 infection of cells with high amount of αvβ3 integrin does not involve clathrin but is caveolin-1-dependent. Thus, this study demonstrates that caveolin-1 is limiting factor in αvβ3 integrin-mediated HAdV26 infection. Regardless of αvβ3 integrin expression, HAdV26 infection involves dynamin-2. Our data provide for the first-time description of HAdV26 cell entry pathway, hence increase our knowledge of HAdV26 infection. Knowing that functionality of adenovirus vector is influenced by its cell entry pathway and intracellular trafficking, our results will contribute to better understanding of HAdV26 immunogenicity and antigen presentation when used as vaccine vector.

**IMPORTANCE** In order to fulfill its role as a vector, adenovirus needs to successfully deliver its DNA genome to the host nucleus, a process highly influenced by adenovirus intracellular translocation. Thus, cell entry pathway and intracellular trafficking determine functionality of human adenovirus-based vectors. Endocytosis of HAdV26, currently extensively studied as a vaccine vector, has not been described so far. We present here that HAdV26 infection of human epithelial cells with high expression of αvβ3 integrin, one of the putative HAdV26 receptors, is caveolin-1- and partially dynamin-2-dependent. Since caveolin containing domains provide a unique environment for specific signaling events and participate in inflammatory signaling one can imagine that directing HAdV26 cell entry toward caveolin-1-mediate pathway might play role in immunogenicity of this virus. Therefore, our results contribute to better understanding of HAdV26 infection pathway, hence, can be helpful in explaining induction of immune response and antigen presentation by HAdV26-based vaccine vector.

## INTRODUCTION

Replication deficient vectors based on human adenovirus type 26 (HAdV26) are extensively studied as a platform for vaccine development (1) and very recently two HAdV26 based vaccines received marketing authorization from European Union: vaccine against Ebola (2) and vaccine against COVID-19 (3). Yet, there are still many unknowns regarding molecular mechanisms involved in the interactions of HAdV26 and host cells during infection, with HAdV26 endocytosis being one of them.

Human adenoviruses (HAdV) are divided into seven subgroups (A–G), with HAdV26 belonging to subgroup D. Although HAdV26 was isolated already in 1961 (4), its natural tropism and cell entry pathway are rather ambiguous. It has been described so far that HAdV26 can use CD46 (5–7), CAR (8), αv integrins (6), scavenger receptor SR-A6 (9), sialic acid (10), and αvβ3 integrin (11) as a receptor in cell infection, indicating that HAdV26 receptor usage could be cell-specific.

Initial binding of HAdV to the primary receptor followed by interaction between RGD domain from the penton base and αv integrins on cell surface triggers signals for HAdV entry into the cell by receptor-mediated endocytosis (12). To date, clathrin-mediated, lipid raft/caveolae-mediated endocytosis and macropinocytosis have been shown to be involved in HAdV entry. Namely, HAdV type 2 (HAdV2) and type 5 (HAdV5) from subgroup C use clathrin-mediated endocytosis (13, 14), HAdV type 37 (HAdV37) from subgroup D uses clathrin- and caveolin-mediated endocytosis (15, 16), whereas HAdVs belonging to subgroup B type 3 (HAdV3) and 35 (HAdV35) use macropinocytosis (17, 18). An essential component of vesicle formation in receptor-mediated endocytosis is dynamin and as such is involved in clathrin- and caveolin-mediated endocytosis, but not in macropinocytosis (19). Role of dynamin in HAdV cell entry has been reported for HAdV5 (20), HAdV2 (21), and HAdV37 (15, 22). Cell entry pathway and intracellular trafficking determine functionality of HAdV based vector (23). In the context of vaccine vector, cell entry pathway can also modulate antigen presentation, thus influence vaccine efficacy. So far it has been suggested that HAdV26 accumulates in late endosomes 2 to 8 h postinfection (24), however more thorough description of HAdV26 endocytosis and intracellular trafficking is still missing.

Previously, we reported that αvβ3 integrin is required for efficient infection of epithelial cells with HAdV26 (11). Here we examined the role of dynamin-2, clathrin, and caveolin-1 in HAdV26 infection and we showed that HAdV26 infection of A549 cell line involves dynamin-2 and clathrin but is caveolin-1-independent. We presented that downregulation of clathrin in A549 cells increased HAdV26 infection by increasing expression of αvβ3 integrin, while inhibition of clathrin-coated pits disabled HAdV26 transport through the cytoplasm, indicating that in A549 cells with low expression of αvβ3 integrin HAdV26 infection can be clathrin-mediated. Finally, by examining in more detail the role of αvβ3 integrin in HAdV26 cell entry, we have shown that αvβ3 integrin-mediated HAdV26 infection involves dynamin-2 and is caveolin-1-dependent. Our data describe for the first time cell entry pathway of HAdV26, currently used as one of the important vaccine vectors. As such, our results contribute to better understanding of HAdV26 infection pathway in general, hence can be helpful in explaining induction of immune response and antigen presentation when HAdV26 is used as a vaccine vector.

## RESULTS

### HAdV26 infection in the A549 cell line is partially dynamin-2-dependent.

It has been shown that internalization and transduction efficiency of HAdV2 (13) and HAdV5 (18) depend on dynamin-2, while direct involvement of dynamin-2 in HAdV intracellular trafficking was reported for HAdV37 (22). Therefore, we wanted to examine the role of dynamin-2 in HAdV26 cell entry.

**FIG 1:**
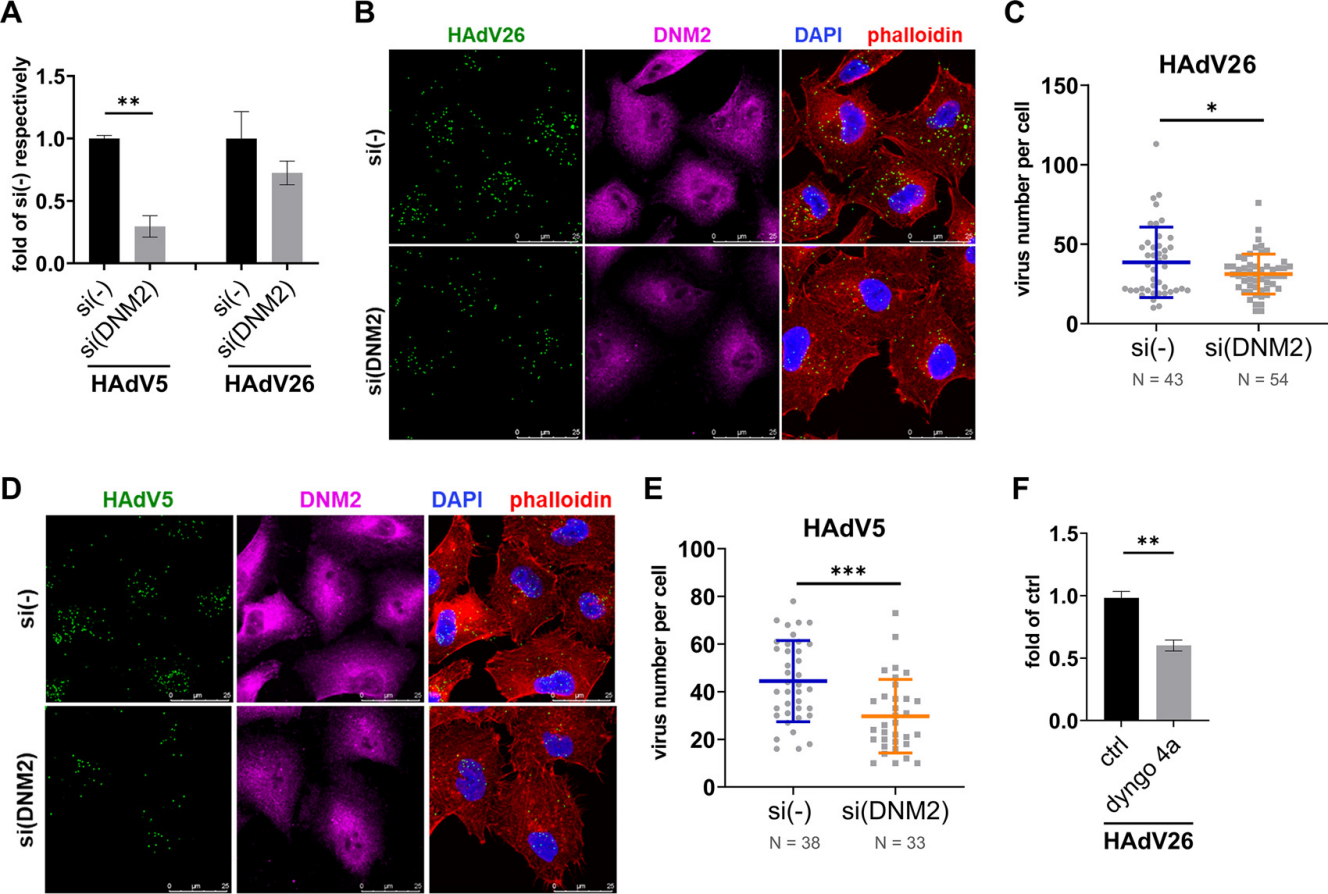
HAdV26 cell infection involves dynamin-2 (DNM2). (A) Transduction efficiency of HAdV5 and HAdV26 in A549 cells after downregulating DMN2 with specific siRNA. (B) Internalization of Alexa Fluor 488-labeled HAdV26 and (D) Alexa Fluor 488-labeled HAdV5 in A549 cells after downregulating DNM2 with specific siRNA. The images are maximum projections of confocal stacks. Representative confocal images are shown (scale bar = 25 μm). (C)/(E) Quantification of (B)/(D) shown as number of viral particles per cell. (F) Transduction efficiency of HAdV26 in A549 cells after treatment with dyngo 4a, inhibitor of DNM2. Data in A, C, E and F are presented as mean ± SD from three independent experiments in duplicates or triplicates. *, *P* < 0.05; **, *P* < 0.01; ***, *P* < 0.001.

We decreased expression of dynamin-2 by using specific siRNA and examined how decreased expression of dynamin-2 influences HAdV26 transduction efficiency and cell internalization. Transfection of A549 cells with dynamin-2 specific siRNA decreased dynamin-2 expression down to 0.23 compared to A549 cells transfected with control siRNA (Fig. S1 in the supplemental material). Downregulating dynamin-2 in A549 cells decreased transduction efficiency of HAdV5 (down to 0.30) and HAdV26 (down to 0.72) (Fig. 1A), as well as internalization of fluorescently labeled HAdV5 (down to 0.67) and HAdV26 (down to 0.81) (Fig. 1B–E), indicating that HAdV26 enters cells via dynamin-2-dependent endocytosis. To further confirm involvement of dynamin-2 on the infection of HAdV26, the transduction efficiency of HAdV26 in A549 was examined after treating cells with dyngo 4a, a specific inhibitor of dynamin-2-dependent endocytosis (25). Dyngo 4a treatment decreased transduction efficiency of HAdV26 by almost 50% compared to untreated cells (Fig. 1F).

Together these results suggest that endocytosis of HAdV26 as well as HAdV5 in A549 cell line requires dynamin-2. Because dynamin-2 is involved in both clathrin- and caveolin-1-mediated endocytosis, our next step was to investigate the role of clathrin and caveolin-1 in HAdV26 cell entry.

### Increased HAdV26 infection in A549 cells with decreased expression of clathrin is due to increased expression of αvβ3 integrin.

Use of clathrin-mediated endocytosis was reported for HAdV2 (14) and HAdV5 (13), but also for non-CAR-binding HAdV37 whose cellular entry in human corneal epithelial cells occurs primarily by clathrin-mediated but dynamin-independent endocytosis (15).

**FIG 2:**
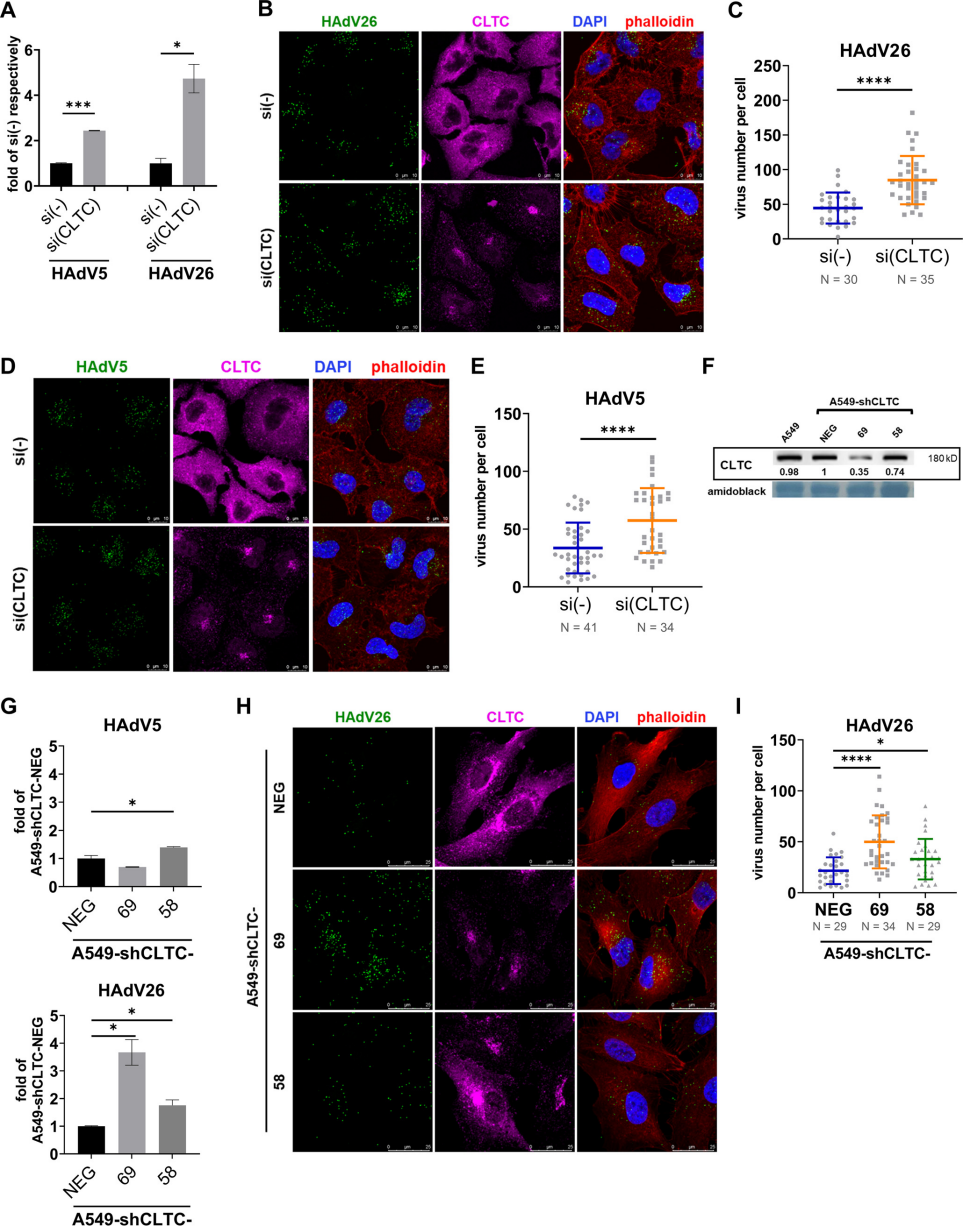
Decreased expression of clathrin (CLTC) increase HAdV26 cell infection. (A) Transduction efficiency of HAdV5 and HAdV26 in A549 cells after downregulating CLTC with specific siRNA. (B) Internalization of Alexa Fluor 488-labeled HAdV26 and (D) Alexa Fluor 488-labeled HAdV5 in A549 cells after dowregulating CLTC with specific siRNA. The images are maximum projections of confocal stacks. Representative confocal images are shown (scale bar = 10 μm). (C,E) Quantification of (B)/(D) shown as number of viral particles per cell. (F) Expression of clathrin in A549 cell clones obtained by stable transfection of A549 cells with a plasmid containing shCLTC. The numbers below the bands represent protein expression in a sample of particular cell line relative to the expression in A549 that was set as 1. (G) Transduction efficiency of HAdV5 and HAdV26 in A549 cells and A549-shCLTC clones with decreased expression of CLTC. (H) Internalization of Alexa Fluor 488-labeled HAdV26 in A549-shCLTC clones with decreased expression of CLTC. The images are maximum projections of confocal stacks. Representative confocal images are shown (scale bar = 25 μm). (I) Quantification of (F) shown as number of viral particles per cell. Data In A, C, E, G and I are presented as mean ± SD from three independent experiments in duplicates or triplicates. *, *P* < 0.05; ***, *P* < 0.001; ****, *P* < 0.000.1.

To study role of clathrin-mediated endocytosis in HAdV26 cell entry we decreased expression of clathrin by means of specific siRNA. Transfection of clathrin heavy chain 1 (CLTC) specific siRNA decreased expression of clathrin down to 0.35 compared to A549 cells transfected with control siRNA (Fig. S1 in the supplemental material). Downregulating clathrin in A549 cells significantly increased HAdV26 transduction efficiency (4.7-fold) as well as internalization (1.9-fold) (Fig. 2A to C). Unexpectedly, silencing of clathrin increased transduction efficiency (2.4-fold) and internalization (1.7-fold) of HAdV5 (Fig. 2A, D, E), which is not consistent with the literature where it has been shown that downregulating clathrin in HeLa cells decreased transduction efficiency of HAdV5 (13).

**FIG 3:**
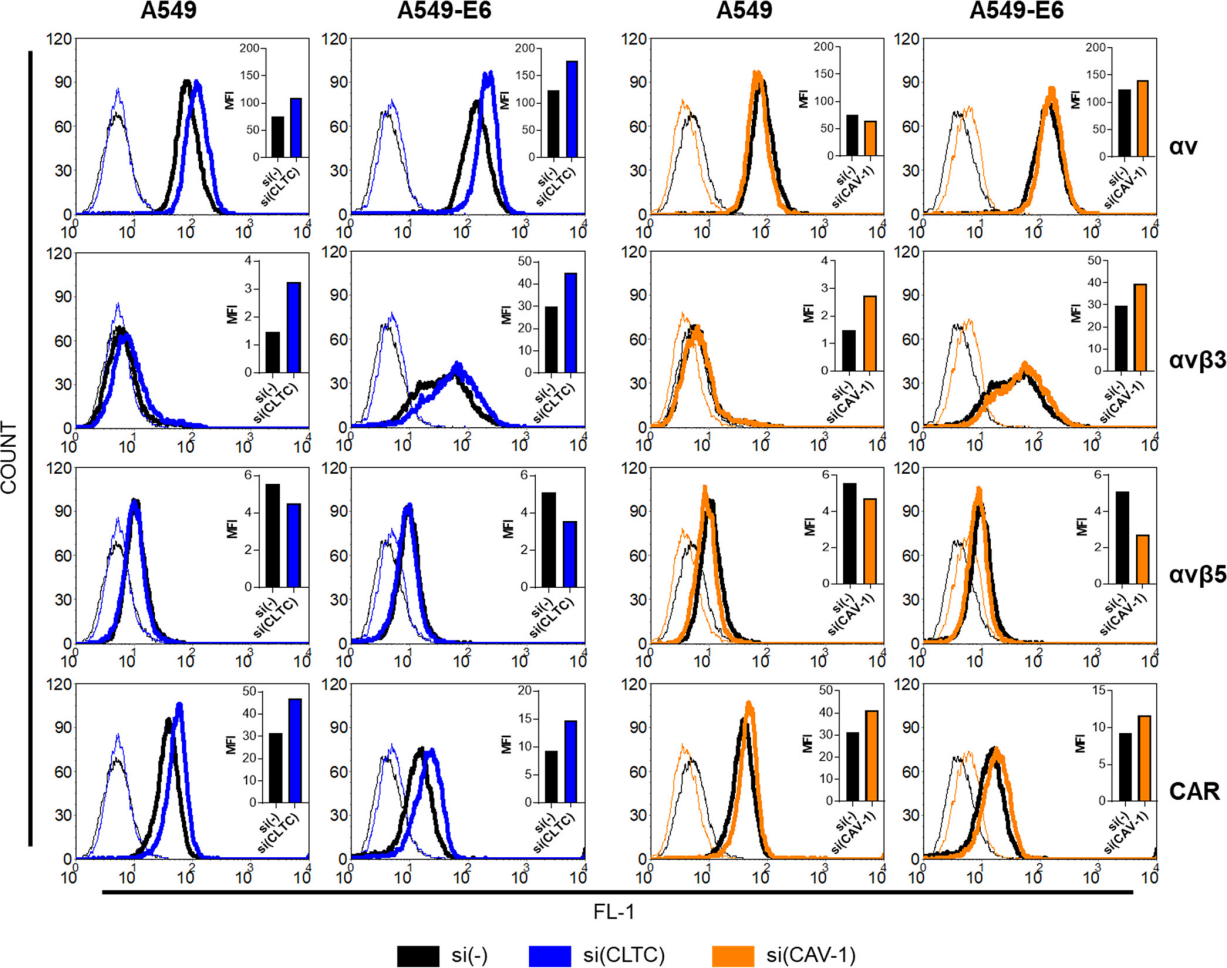
Decreased expression of clathrin (CLTC) or caveolin-1 (CAV-1) changes expression of integrins and CAR. Expression of αv, αvβ3 and αvβ5 integrins and CAR on surface of A549 and A549-E6 cells was determined by flow cytometry 48 h after downregulating CLTC or CAV-1 with specific siRNA. Thin lines histograms represent isotype controls and bold lines the expression of the corresponding protein. Comparison of geometric MFIs for corresponding histograms are shown in upper right corners. The representative data of two independent experiments which yielded comparable results are shown (*n* = 2).

Downregulating key proteins of a particular type of endocytosis can disrupt the recycling of molecules present on the cell surface. Therefore, the effect of downregulated clathrin on the cell surface expression of known adenoviral receptors was investigated. Downregulation of clathrin increased the expression of CAR, αv and αvβ3 integrins in the A549 cells, while the amount of αvβ5 integrin on the cell surface was not significantly altered (Fig. 3).

HAdV26 infection was studied also in clones with stably downregulated clathrin. We isolated two A549 clones stably transfected with a plasmid expressing shRNA specific for clathrin: A549-shCLTC-58 and -69, as well as one control clone transfected with plasmid backbone: A549-shCLTC-NEG. Western blot analysis showed that the amount of clathrin in the A549-shCLTC-NEG clone was the same as in A549 cells, whereas A549-shCLTC-58 and -69 clones showed decreased expression of clathrin (0.74 and 0.35 respectively compared to clathrin expression in A549-shCLTC-NEG clone) (Fig. 2F). Consistent with the results of downregulating clathrin by transient transfection using siRNA, clones with stably decreased expression of clathrin show significantly increased transduction efficiency and internalization of HAdV26 compared to A549-shCLTC-NEG (Fig. 2G–I). In A549-shCLTC-58, transduction efficiency with HAdV26 was increased 1.7-fold, and in A549-shCLTC-59 3.7-fold compared to A549-shCLTC-NEG. Interestingly, in A549-shCLTC-58, transduction efficiency with HAdV5 was also slightly increased (1.4-fold), while in A549-shCLTC-69 was slightly decreased (down to 0.70) compared to A549-shCLTC-NEG (Fig. 2G). HAdV26 binding in A549-shCLTC-58 and -69 was increased in comparison to A549 (Fig. S2A in the supplemental material), which is in line with transduction efficiency in these cell clones. Next, we measured surface expression of αv, αvβ3 and αvβ5 integrin, and CAR in A549-shCLTC-58 and -69 cell clones (Fig. S3). Compared to the A549-shCLTC-NEG clone, both clones with stably decreased clathrin had a significantly increased amount of αvβ3 integrin which is consistent with increased entry of HAdV26. A549-shCLTC-58 had no altered amount of CAR, while A549-shCLTC-69 had a reduced amount of CAR expression relative to A549-shCLTC-NEG. This reduction is consistent with the decreased transduction efficiency of HAdV5 that we saw in A549-shCLTC-69.

All together we can conclude that downregulating clathrin significantly increases HAdV26 and HAdV5 entry by increasing expression of their corresponding receptor on the surface of the A549 cells, i.e., αvβ3 integrin for HAdV26 (11), and CAR (26) and αv/αvβ3 integrins (27) for HAdV5.

### Inhibition of clathrin-coated pits formation significantly increases infection with HAdV26.

**FIG 4:**
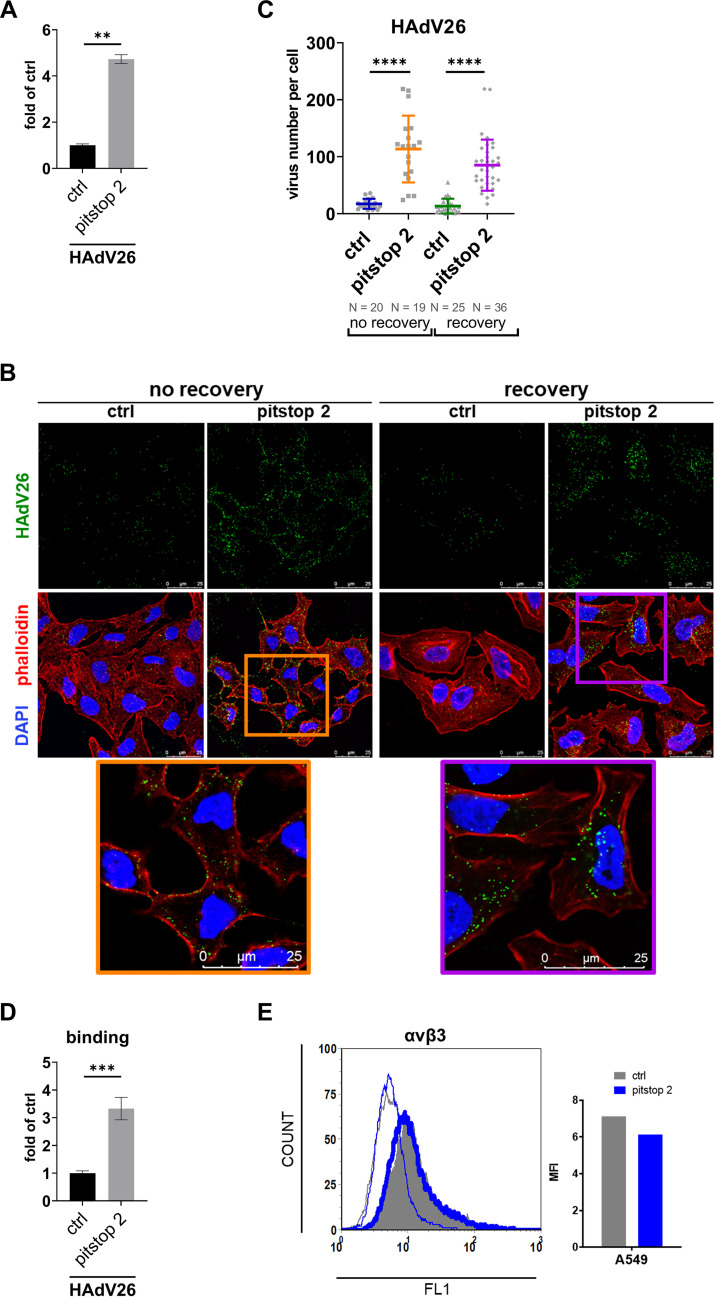
Inhibition of clathrin-mediated endocytosis stalls HAdV26 intracellular trafficking and increases infection with HAdV26 without changing expression of αvβ3 integrin. (A) Transduction efficiency of HAdV26 in A549 cells after treatment with pitstop 2, inhibitor of clathrin-mediated endocytosis. Pitstop was removed from the cells 2 h postinfection. (B) Internalization of Alexa Fluor 488-labeled HAdV26 in A549 cells after treatment with pitstop 2. The images are maximum projections of confocal stacks. Representative confocal images are shown (scale bar = 25 μm). (C) Quantification of (B) shown as number of viral particles per cell. (D) Binding of HAdV26 in A549 cells after treatment with pitstop 2. (E) Expression of αvβ3 integrins on the surface of A549 cells after treatment with pitstop 2. Results from left panel were presented as comparisons of geometric MFIs on the right panel. Data in A, C and D data are presented as mean ± SD from three independent experiments in duplicates or triplicates. **, *P* < 0.01; ***, *P* < 0.001; ****, *P* < 0.000.1.

To further examine the involvement of clathrin-mediated endocytosis in the cell entry of HAdV26, the transduction efficiency of HAdV26 was examined in A549 cells treated with pitstop 2, a specific inhibitor of clathrin-dependent endocytosis. Pitstop 2 treatment increased transduction efficiency of HAdV26 almost 5-fold compared to untreated cells (Fig. 4A) (n.b. pitstop 2 was removed from the cells 2 h postinfection). As far as internalization is concerned, treatment with pitstop 2 stalled HAdV26 in the proximity of the cell membrane which indicates that the use of pitstop 2 stopped intracellular trafficking of HAdV26. When we removed pitstop 2 from incubation medium in the recovery condition, HAdV26 was found scattered over the cytoplasm with most of the viral particles in the perinuclear region, indicating that removal of pitstop 2 allows reconstitution of HAdV26 intracellular trafficking (Fig. 4B). Of note, pitstop 2 action is reversible, with clathrin-mediated endocytosis being fully restored after a 30 min drug washout (28).

By counting fluorescently labeled HAdV26 that entered the cell, we saw that internalization of HAdV26 is increased in A549 cells treated with pitstop 2 (6.6- in no recovery and 6.4-fold in recovery condition; Fig. 4C). This increase is due to the increased binding of HAdV26 in pitstop 2 treated cells. Namely, HAdV26 showed 3.2-fold increased binding after treatment with pitstop 2 (Fig. 4D). Both increased binding and internalization of HAdV26 in pitstop 2 treatment are consistent with increased transduction efficiency obtained after the same treatment thus we conclude that increased transduction efficiency of HAdV26 after pitstop 2 treatment is direct consequence of increased binding and internalization. Contrary to our experiment with clathrin knockdown where we clearly demonstrated that decreased expression of clathrin increased expression of αvβ3, pitstop 2 did not alter expression of αvβ3 integrin on cell surface (Fig. 4E). Exact reason(s) for increased binding and cell entry of HAdV26 in pitstop 2 treated A549 cells remains to be investigated. We can conclude that role of clathrin is evident in two steps of HAdV26 cell entry: i) the amount of αvβ3 integrin available on cell surface and ii) intracellular trafficking of HAdV26 particles.

### HAdV26 infection in A549 cell line expressing low amount of αvβ3 integrin is caveolin-1-independent.

Use of caveolae-mediated cell entry was reported for chimeric adenoviral vectors harboring fibers constituted of the N-terminal domain of HAdV2 and the knob domain of a bovine adenovirus BAdV4 (29), but also for HAdV37 which enters human keratocytes through caveolae (16).

**FIG 5:**
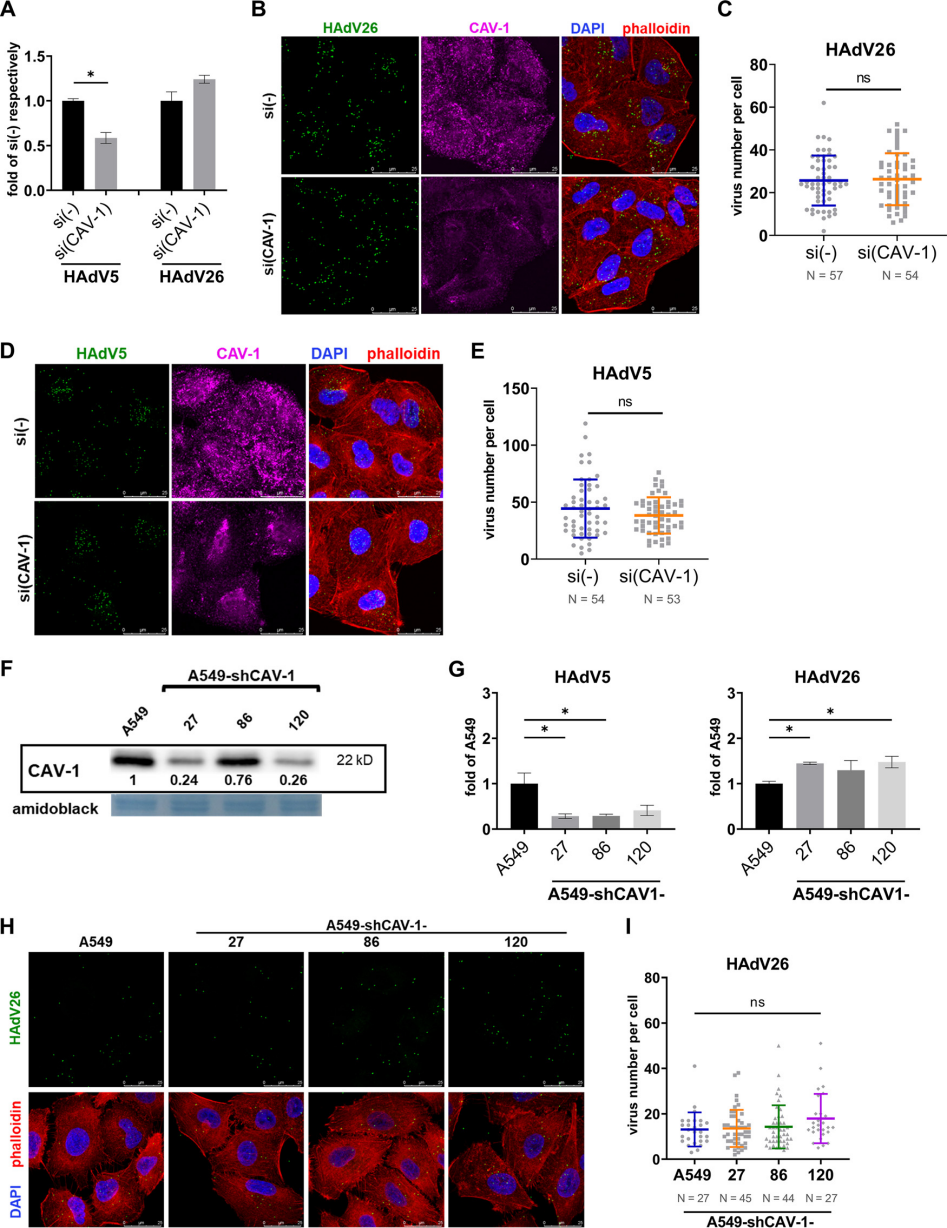
Decreased expression of caveolin-1 (CAV-1) has small impact on HAdV26 infection in cells with low expression of αvβ3 integrin. (A) Transduction efficiency of HAdV5 and HAdV26 and in A549 cells after downregulating CAV-1 with specific siRNA. (B) Internalization of Alexa Fluor 488-labeled HAdV26 and (D) Alexa Fluor 488-labeled HAdV5 in A549 cells after downregulating CAV-1 with specific siRNA. The images are maximum projections of confocal stacks. Representative confocal images are shown (scale bar = 25 μm). (C)/(E) Quantification of (B)/(D) shown as number of viral particles per cell. (F) Expression of CAV-1 in A549 cell clones obtained by stable transfection of A549 cells with a plasmid containing shCAV-1. The numbers below the bands represent protein expression in a sample of particular cell line relative to the expression in A549 that was set as 1. (G) Transduction efficiency of HAdV5 and HAdV26 in A549 cells and A549-shCAV-1 clones with decreased expression of CAV-1. (H) Internalization of Alexa Fluor 488-labeled HAdV26 in A549 and A549-shCAV-1 clones with decreased expression of CAV-1. The images are maximum projections of confocal stacks. Representative confocal images are shown (scale bar = 25 μm). (I) Quantification of (F) shown as number of viral particles per cell. Data in A, C, E, G and I are presented as mean ± SD from three independent experiments in duplicates or triplicates. *, *P* < 0.05.

To study role of caveolin-1 in HAdV26 infection pathway we decreased its expression by transfection with specific siRNA prior infection with HAdV26. Amount of caveolin-1 in A549 cells transfected with caveolin-1 siRNA was decreased down to 0.38 compared to A549 cells transfected with control siRNA (Fig. S1 in the supplemental material). Downregulating caveolin-1 by siRNA slightly increased transduction efficiency of HAdV26 (1.2-fold) and decreased transduction efficiency of HAdV5 (down to 0.58) (Fig. 5A). Downregulation of caveolin-1 did not change the number of successfully internalized HAdV26 (Fig. 5B and C) and HAdV5 (Fig. 5D, E), which is consistent with the transduction efficiency of HAdV26, but not for HAdV5 where transduction efficiency is decreased after silencing caveolin-1 (Fig. 5A). Determining the number of HAdV particles that successful internalized into cells does not provide data on the successful entry of the HAdV genome into the nucleus. It is possible that downregulation of caveolin-1 does not alter the number of HAdV5 particles that are successful internalized into the cell, but has an impact on their successful intracellular trafficking to the nucleus and/or successful delivery of viral DNA into the cell nucleus, hence transduction efficiency.

Role of caveolin-1 in HAdV26 cell infection was additionally assessed in cell clones with stably downregulated caveolin-1. We isolated three A549 clones stably transfected with a plasmid expressing shRNA specific for caveolin-1: A549-shCAV-1-27, -86, and -120, which have differently decreased expression of caveolin-1 compared with A549 cells (ranging 0.24–0.76, respectively, compared with clathrin expression in A549) (Fig. 5F). Transduction efficiency and internalization of HAdV26 in A549-shCAV-1 clones was either slightly increased or unchanged compared to A549 **(**Fig. 5G to I), which is consistent with the results we obtained when we silenced caveolin-1 by transient transfection of siRNA. Contrary to transduction efficiency, HAdV26 binding in A549-shCAV-1-27, -86 and -120 was increased in comparison to A549 (Fig. S2B). As in A549 cells transiently transfected with caveolin-1 siRNA, transduction efficiency of HAdV5 in A549-shCAV-1 clones was decreased by more than 50% compared to A549 cells (Fig. 5G). Expression of CAR and αvβ5 integrins in A549-shCAV-1 clones is significantly reduced compared to A549 cells (Fig. S4), which can account for decreased HAdV5 transduction efficiency. Namely, αvβ5 integrin is important for successful release of HAdV5 from endosome (30). A549-shCAV-1 clones have highly increased amount of αvβ3 integrin expressed on the surface, yet this surprisingly didn’t lead to significantly higher HAdV26 cell entry or transduction efficiency, as we saw in A549-E6 cell clone of A549 cells with overexpression of αvβ3 integrin (11) or in this study with cell clone A549-shCLTC-69. Thus, we hypothesized that caveolin-1 could be limiting factor in αvβ3 integrin-mediated HAdV26 cell infection.

### HAdV26 infection in the A549 cell clone with increased expression of αvβ3 integrin is caveolin-1-dependent.

**FIG 6:**
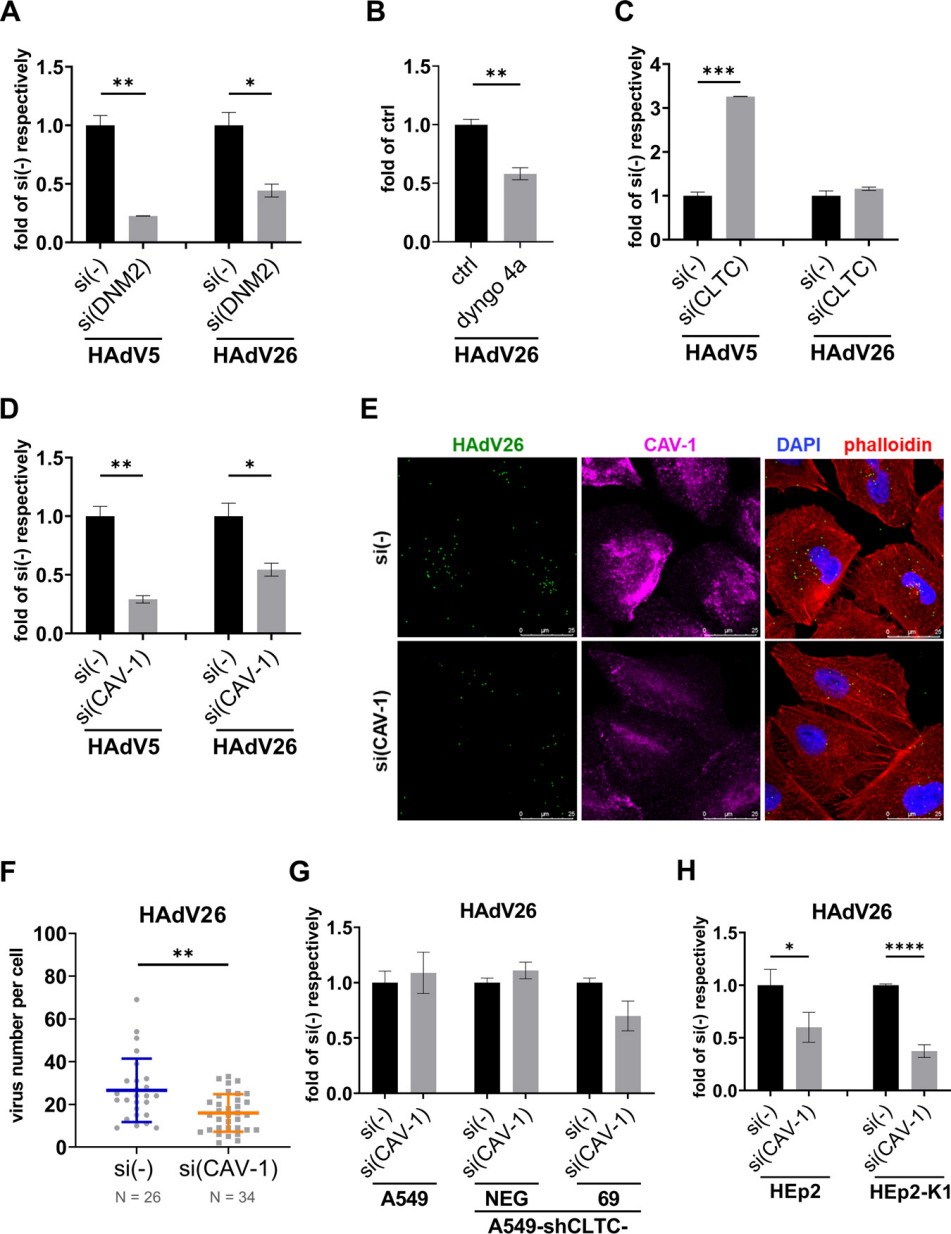
αvβ3 integrin-mediated HAdV26 infection is caveolin-1-dependent. (A) Transduction efficiency of HAdV5 and HAdV26 and in A549-E6 cells after downregulating DNM2 with specific siRNA. (B) Transduction efficiency of HAdV26 in A549-E6 cells after treatment with dyngo 4a. (C) Transduction efficiency of HAdV5 and HAdV26 and in A549-E6 cells after downregulating CLTC with specific siRNA. (D) Transduction efficiency of HAdV5 and HAdV26 and in A549-E6 cells after downregulating CAV-1 with specific siRNA. (E) Internalization of Alexa Fluor 488-labeled HAdV26 in A549-E6 cells after downregulating CAV-1 with specific siRNA. The images are maximum projections of confocal stacks. Representative confocal images are shown (scale bar = 25 μm). (F) Quantification of (E) shown as number of viral particles per cell. (G) Transduction efficiency of HAdV26 in A549, A549-shCLTC-NEG and -69 cells after silencing CAV-1 with specific siRNA. (H) Transduction efficiency of HAdV26 in HEp2 and HEp2-K1 cells after silencing CAV-1 with specific siRNA. Data in A–D and F–H are presented as mean ± SD from three independent experiments in duplicates or triplicates. *, *P* < 0.05; **, *P* < 0.01; ***, *P* < 0.001; ****, *P* < 0.000.1.

It has been shown that αvβ3 integrin can be involved in clathrin- (31, 32) and caveolin-mediated endocytosis (33). To further examine connection between αvβ3 integrin and HAdV26 endocytosis we used previously constructed A549-E6 clone which has stably increased expression of αv integrin, especially αvβ3 integrin and due to that exhibit better cell entry and transduction efficiency with HAdV26 (11). Here we showed that transduction efficiency of HAdV26 in A549-E6 is dynamin-dependent since dynamin-2 downregulation and dyngo 4a treatment significantly decreased transduction efficiency of HAdV26 (down to 0.44 and 0.58) (Fig. 6A and B). To our surprise, downregulating clathrin did not affect HAdV26 transduction efficiency (Fig. 6C) although it increased the expression of αvβ3 integrin (Fig. 3). We assume that there is a certain threshold in αvβ3 integrin expression needed for successful transduction with HAdV26 which is already reached in A549-E6, thus further increase in αvβ3 integrin will have only incremental effect on HAdV26 transduction efficiency in this cell clone. Transduction efficiency of HAdV5 in A549-E6 clone is decreased after downregulating dynamin-2 (down to 0.22) or caveolin-1 (down to 0.29) and increased after silencing of clathrin (3.3-fold) (Fig. 6A, C, D), following the same pattern as in A549 cells (Fig. 1A, 2A, 5A).

Next, we downregulated caveolin-1 in A549-E6 cells and determined transduction efficiency and internalization of HAdV26. We observed that decreased expression of caveolin-1 decreased transduction efficiency (Fig. 6D) and internalization (Fig. 6E and F) of HAdV26 by 50%, although αvβ3 integrin expression remained unchanged (Fig. 3), indicating once again involvement of caveolin-1-dependent endocytosis in αvβ3 integrin-mediated HAdV26 infection. To finally test this hypothesis, we used A549-shCLTC-69 cell clone which has stably decreased clathrin expression, increased αvβ3 integrin expression and due to that increased HAdV26 infection. We presumed that silencing of caveolin-1 in A549-shCLTC-69 should reduce the increased HAdV26 entry caused by increased integrin αvβ3 integrin expression. Thus, we transfected A549-shCLTC-69 with caveolin-1 siRNA and measured transduction efficiency of HAdV26. Decreased expression of caveolin-1 in A549-shCLTC-69 cells decreased transduction efficiency of HAdV26 by 30% compared to A549-shCLTC-69 transfected with control siRNA. Downregulation of caveolin-1 had no effect on HAdV26 transduction efficiency in A549 or A549-shCLTC-NEG (Fig. 6G). Importance of caveolin-1 in αvβ3 mediated HAdV26 transduction efficiency was further corroborated in additional cell model, namely, HEp2-K1, cell clone of HEp2 cells with increased expression of αvβ3 integrin (34). Downregulation of caveolin-1 (Fig. S1 in the supplemental material) significantly decreased HAdV26 transduction efficiency in HEp2-K1 cells (Fig. 6H). Of note, HAdV26 transduction efficiency is 3.7-fold higher in HEp2-K1 in comparison to parental HEp2 αvβ3 integrin negative cells (Fig. S5). All together these results confirmed our hypothesis and suggest that using αvβ3 integrin as a receptor directs HAdV26 toward caveolin-1-mediated endocytosis.

## DISCUSSION

Because of its low seroprevalence in humans as well as induction of favorable immune response to transgene, HAdV26 has been recognized as a promising platform for vaccine vector development and is studied in number of completed or ongoing clinical studies (1). Nevertheless, basic biology of this virus is still insufficiently known. Namely, there are no reports describing cell entry pathway, i.e., endocytosis of HAdV26. Therefore, in this study we investigated role of dynamin-2, clathrin and caveolin-1 in HAdV26 infection in A549 cell line, which is commonly used for adenovirus research, and previously constructed clone A549-E6 with stably increased expression of αvβ3 integrin that allows better infection with HAdV26 compared to parental A549 cells (11).

**FIG 7:**
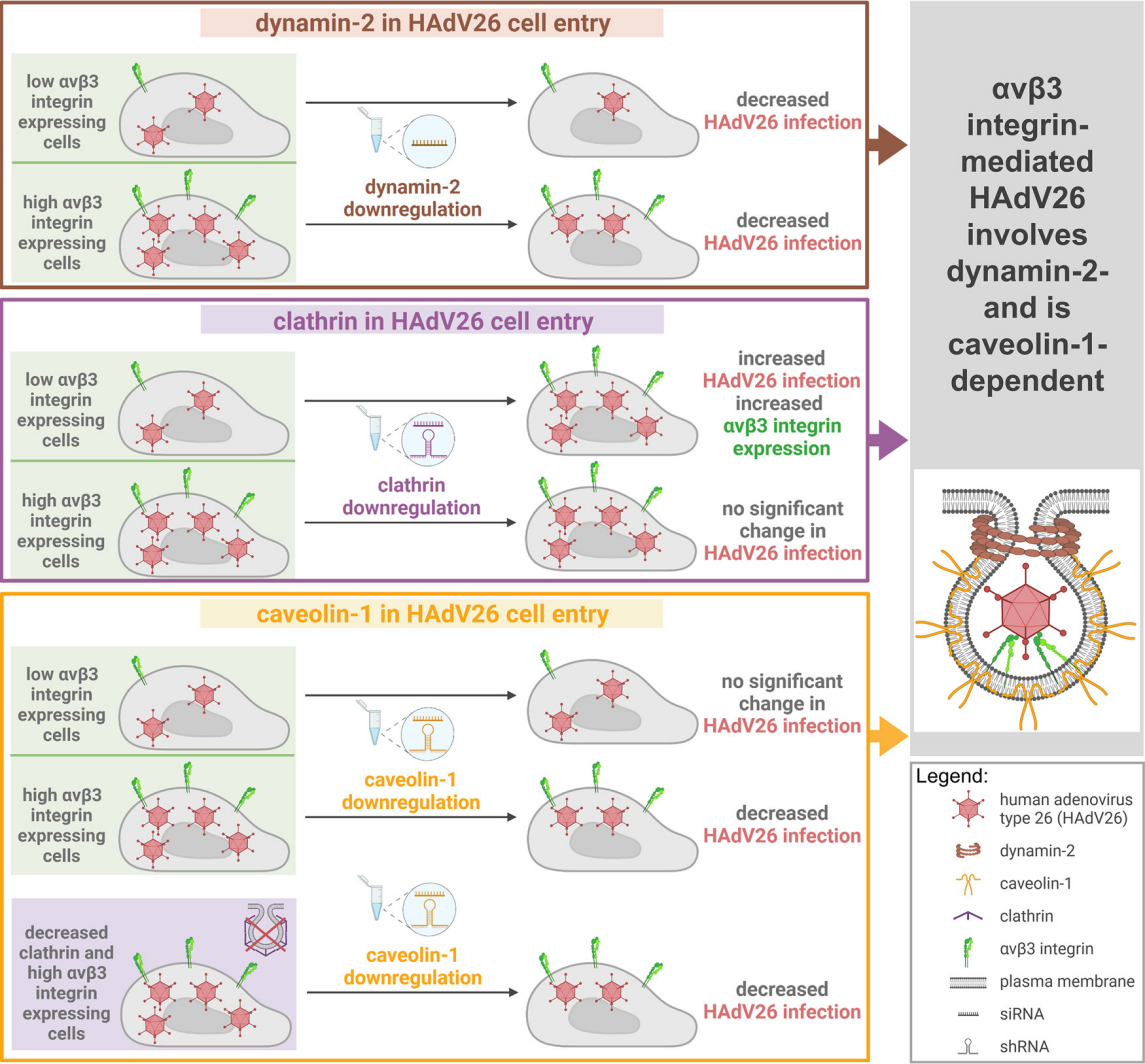
A model for the role of dynamin-2, clathrin and caveolin-1 in HAdV26 cell entry. For the infection of αvβ3 integrin positive human epithelial cells HAdV26 uses caveolin-1-mediated endocytosis. Regardless of αvβ3 integrin, HAdV26 infection involves dynamin-2. Role of clathrin in HAdV26 infection is indirect, namely, downregulation of clathrin increases expression of αvβ3 integrin and subsequently transduction efficiency of HAdV26. Created with BioRender.com.

We have shown here that HAdV26 infection of A549 cells which have very low amount of αvβ3 integrin involves dynamin-2 and is caveolin-1-independent, while infection of A549-E6 cells which express high amount of αvβ3 integrin involves dynamin-2- and is caveolin-1-dependent, indicating that caveolin-1 is limiting factor in αvβ3 integrin-mediated HAdV26 infection. HAdV26 infection of A549 cells which express low amount of αvβ3 involves clathrin but rather on the level of the receptor, i.e., downregulating clathrin caused increased expression of αvβ3 integrin which subsequently increased infection with HAdV26. Our data provide insight into quite interesting nature of HAdV26 infection pathway suggesting that depending on the receptor status this virus can enter the cell in different manner which can be independent of dynamin-2, clathrin and/or caveolin-1. A model explaining role of dynamin-2, clathrin and caveolin-1 in HAdV26 cell entry is presented on Fig. 7.

During the endocytic pathway of HAdV after virus binding to receptors on the cell surface, plasma membrane invagination occurs. Release of the newly formed endocytic vesicle into the cytosol involves scission factors, often dynamin-2 (35). Here we demonstrated that HAdV26 infection involves dynamin-2 regardless of cell lines used for analysis, i.e., decreased expression or inhibited function of dynamin-2 decreased both internalization and transduction efficiency of HAdV26. In this respect HAdV26 is more similar to HAdV2 and HAdV5 from subgroup C, than HAdV37 which, like HAdV26, belongs to adenovirus subgroup D. Namely, interfering with dynamin-2 decreased transduction efficiency of HAdV2 and HAdV5 (20, 21), while dynamin-2 knockdown led to increased HAdV37 DNA nuclear entry and viral replication (22).

By interfering with clathrin-mediated endocytosis, either by decreasing clathrin expression or inhibiting clathrin-coated pits, we have shown that clathrin has a dual role in HAdV26 infection. Downregulation of clathrin increases the amount of αvβ3 integrin on the cell surface which serves as a receptor for HAdV26 (11), thereby increasing both cell internalization and transduction efficiency with HAdV26. We presume that change in expression of αvβ3 integrin is most likely due to disorder in αvβ3 integrin recycling since it has been reported that clathrin-mediated endocytic machinery is involved in the endocytosis of pentapeptide cyclic RGD-integrin-αvβ3 clusters (32). In the case of inhibition of clathrin-mediated endocytosis, after pitstop 2 treatment, there are no changes in αvβ3 integrin expression, and yet there is a strong increase in binding and transduction efficiency of HAdV26. Treatment with pitstop 2 stopped HAdV26 in close proximity of the plasma membrane, which would mean that pitstop 2 does not restrict binding or entry of HAdV26 but prevents intracellular trafficking of HAdV26. Since pitstop 2 inhibits the binding of clathrin to the already formed pit (36), it is possible that i) pit formation due to HAdV26 binding resulted in activation and accumulation of αvβ3 integrin, which due to inhibition of later steps in the formation of mature endocytic vesicles can bind more HAdV26, or ii) inhibition of clathrin leaves potential clathrin-coated vesicle open and accessible to accumulation of a large number of viruses which, at the moment when the inhibitor is removed from the cells, continue their traffic to the nucleus. Increased binding and transduction efficiency of HAdV26 after inhibiting clathrin-coated pits could also be indicative of HAdV26 ability to use other receptors than αvβ3 integrin. Each of these hypotheses still needs to be tested experimentally. However, it can be concluded that clathrin is involved in HAdV26 cell entry but successful HAdV26 infection is clathrin-independent. Role of clathrin-mediated endocytosis has been shown for HAdV2 and HAdV5 that bind CAR, in contrast to HAdV37 which for infection of human corneal cells uses αvβ1 and α3β1 integrins (37). While HAdV2 and HAdV5 showed canonical clathrin-mediated endocytosis, which includes clathrin adaptors, dynamin, early endosome, and endosomal acidification (13, 14, 38), HAdV37 showed no involvement of clathrin adaptor epsin, early endosomal antigen 1 (EEA1), or endosome acidification (15). Research on similar factors for HAdV26 has yet to be done.

Here we have shown that in the settings where HAdV26 can use αvβ3 integrin as a receptor, presence of caveolin-1 is needed for successful infection. Namely, while HAdV26 binding in A549-shCLTC and A549-shCAV1 clones which have increased expression of αvβ3 integrin was comparable (Fig. S2 in the supplemental material), transduction efficiency in A549-shCLTC clones was accordingly increased (Fig. 2G), but in A549-shCAV1 clones (Fig. 5G) was not in the same line as binding, leading us to hypothesize that αvβ3 integrin mediated HAdV26 infection is caveolin-1-dependent. This was further corroborated in αvβ3 integrin positive A549-shCLTC-69 and HEp2-K1, where downregulating caveolin-1 significantly decreased otherwise increased HAdV26 transduction efficiency (Fig. 6G and H). It has been reported that previously discussed HAdV37, belonging to subgroup D as HAdV26, uses a lipid raft-mediated caveolin-1-associated pathway for entry into corneal cells (16). It has been shown that HAdV37 uses αvβ1 and α3β1 integrins for infection of human corneal epithelial cells (37) leading us to speculate that using integrins as receptors can steer HAdVs toward caveolin-mediated cell entry. Usage of caveolae for cell entry was reported also for retargeted HAdVs. Uptake of HAdV5 and pseudotyped HAdV2 with the knob domain of a bovine adenovirus type 4 (BAdV4), HAdV2/BAdV4, through caveolae was shown (29, 39). Nevertheless, the authors suggested that caveolae-dependent entry of HAdV occurs when the clathrin-dependent pathway is not available. In addition, other virus types have been shown to enter different cell lines via different types of endocytosis, such as severe acute respiratory syndrome coronavirus (SARS-CoV), which enters HepG2 and COS7 cells in clathrin-dependent manner, while entering Vero E6 cells is independent of clathrin and caveolae (40, 41). However, it is interesting that the porcine epidemic diarrhea virus (PEDV) enters the same cells by different mechanisms, thus PEDV enters Vero and IPEC-J2 cells via clathrin-, caveolae-, and lipid rafts-mediated pathways (42). Capacity of αvβ3 integrin to dictate virus cell entry was reported also for herpes simplex virus for which was shown that αvβ3 integrin routes this virus to an entry pathway dependent on cholesterol-rich lipid rafts and dynamin-2 (43). Caveolae provide a unique environment for specific signaling events and participate in inflammatory signaling (44) hence one can imagine that directing HAdV26 cell entry toward caveolin-mediate pathway might play role in immunogenicity of this virus.

Clathrin-mediated endocytosis is thought to be the major route of entry of HAdV5 into cells. Yet, we observed that infection with HAdV5 is caveolin-1-dependent and clathrin-independent. In our cell model, downregulation of clathrin increased infection with HAdV5, while downregulation of caveolin-1 decreased infection with HAdV5. HAdV5 infection was highly dependent on CAR expression, and yet in cases where CAR expression was increased (Fig. 3) a downregulation of claveolin-1 decreased HAdV5 infection (Fig. 5A, Fig. 6E). Therefore, we conclude that in our studies, HAdV5 infection was doubly affected, by caveolin-1 and CAR expression. Similar results were observed for HAdV2, another CAR-binding HAdV from subgroup C, which appeared also to use the caveolin-1-dependent pathway for entry into A549 cells (16).

Here we characterized entry mechanism used by HAdV26 to enter epithelial cells. To the best of our knowledge this is the first study describing usage of different HAdV endocytosis pathways in dependence of the receptor availability, but also HAdV26 cell entry in general. Given that HAdV26 has proven to be an extremely important vector used for health purposes in recent years, the data we present provide a better understanding of the basic biology of HAdV26, and therefore offer the possibility of further improving HAdV26-based vectors.

## MATERIALS AND METHODS

### Cells and viruses.

HEK293 (human embryonic kidney; ATCC CRL-1573), HEp2 (human laryngeal carcinoma, ATCC CCL-23), and A549 (human lung carcinoma; ATCC CCL-185) cells were obtained from the American Type Culture Collection (ATCC) and were grown in antibiotic-free DMEM (Sigma-Aldrich, USA) supplemented with 10% (vol/vol) FBS (Sigma-Aldrich, USA) at 37°C with 5% CO2 (vol/vol) in a humidified atmosphere. Cells were regularly screened for mycoplasma presence with Hoechst 33258 (Invitrogen, USA) staining. A549-E6 cell clone (A549 cells with stably increased expression of αv integrin, especially αvβ3 integrin) was previously described (11). HEp2-K1 cell clone (HEp2 cells with stably increased expression of β3 integrin, especially αvβ3 integrin) was previously described (34). The A549 cell line was used for transfection and isolation of stably transfected cell clones (A549-shCLTC and A549-shCAV-1).

Replication-incompetent recombinant adenoviral vectors based on human adenovirus type 26 (HAdV26) or 5 (HAdV5) were previously constructed (5, 45) and have green fluorescent protein (GFP) as a transgene. The vectors were propagated on HEK293 cells and purified by CsCl gradients. CsCl was removed from virus by using PD-10 desalting column (Sephadex G-25M, Amersham Pharmacia Biotech, UK) in PBS according to the manufacturer's protocol. Glycerol was added in final 10% (vol/vol) before aliquots freezing at −80°C. Viral titers were determined as previously described (46).

### Adenovirus labeling.

After purification, adenovirus particles were incubated with a 20-fold molar excess of chemically reactive Alexa Fluor 488 5-TFP (A30005, Thermo Fisher Scientific, USA) for 2 h in the dark at room temperature with gently stirring in 10% glycerol in PBS with 100 nM sodium bicarbonate, pH 7.2. Unbound dye was removed from the labeled virus using Zeba desalting columns (cat. 89889, Thermo Fisher Scientific, USA) with exchange buffer 10% glycerol in PBS according to the manufacturer's protocol. Flow through aliquots containing labeled virus (HAdV-AF488) were stored at −80°C.

### Transduction efficiency assay.

Cells were seeded in 12-well plates at a density of 5 × 10^4^ cells per well in antibiotic-free DMEM supplemented with 10% (vol/vol) FBS, and 24 h later incubated with HAdV vectors at a dose of 10^4^ vp/cell in a total volume of 500 μL of antibiotic-free DMEM supplemented with 0.2% (vol/vol) FBS and incubated at 37°C, 5% CO_2_ for 2 h. The medium was removed and replaced with of antibiotic-free DMEM supplemented with 10% (vol/vol) FBS and cultured for additional 48 h. Cells were detached by trypsin (Sigma-Aldrich, USA), washed twice with PBS and fixed with 1% paraformaldehyde in PBS for 10 min at room temperature. Cells were then washed twice with PBS and resuspended in PBS. Transduction efficiency was measured by flow cytometry using FACSCalibur (BD Biosciences, USA), while cell acquisition was made using BD CellQuest software package (BD Biosciences, USA). Number of acquired event per sample was 10,000. Data were analyzed using FCS Express 3 (De Novo Software, USA), and showed as transduction efficiency corresponding to the geometric mean of the fluorescence intensity (MFI) of GFP signal encoded by adenoviral vectors shown as a value relative to the corresponding control sample.

### Adenovirus binding.

Cells were seeded in 6-well plates at a density of 3 × 10^5^ cells per well in antibiotic-free DMEM supplemented with 10% (vol/vol) FBS, and 24 h later HAdV were added to cells (10^3^ vp/cell) and incubated for 1 h on ice and 5 min at 37°C. Unbound viruses were removed by washing the cells twice with cold trypsin and twice with cold PBS. The cells were then harvested with a cell scraper and pelleted by centrifugation. Total DNA (cellular and viral) was isolated from cell pellets using the QIAamp DNA Blood minikit (Qiagen, Germany) according to the manufacturer's instructions. Viral DNA was quantified by qPCR on 100 ng of total DNA. Viral DNA was detected by qPCR using primers for the CMV sequence (F: TGGGCGGTAGGCGTGTA, R: CGATCTGACGGTTCACTAAACG). The amount of viral DNA was normalized using expression of GAPDH (glyceraldehyde-3-phosphate dehydrogenase; F: AGAACATCATCCCTGCCTCTACTG, R: TGTCGCTGTTGAAGTCAGAGGAGA). qPCR was performed on a StepOnePlus real-time PCR system (Applied Biosystems, USA), using the SybrGreen reagent (Applied Biosystems, USA). qPCR conditions were as follows: initial denaturation for 10 min at 95°C, 40 cycles of 15 s at 95°C and 1 min annealing at 60°C. The StepOne Software (Applied Biosystems, USA) was used to determine Ct values. The GAPDH was used for assesing ΔΔCt, and fold changes were calculated using the standard 2^-ΔΔCt^ method (47).

### Downregulation by siRNA.

The small interfering RNAs (siRNAs) were prepared by dissolving lyophilized siRNAs in nuclease free H_2_O at a concentration of 100 μM. Working solutions were prepared by further dilution in nuclease free H_2_O to a final concentration of 10 μM and stored at −20°C prior use. To downregulate specific receptors, we used the following Silencer Select predesigned siRNAs: DNM2 siRNA (s4212) at 35 nM, CLTC siRNA (s477) at 35 nM, CAV-1 siRNA (s2446) at 25 nM, and scrambled siRNA no. 1 (control siRNA, si[-], catalog no. 4390844) at 35 nM final concentration, all from Ambion (USA). Cells were transfected at a confluence of 60–75% using Lipofectamine RNAiMax reagent (Thermo Fischer Scientific, USA) according to the manufacturer’s protocol. 48 h after transfection the efficiency of downregulation was verified by Western blotting analysis or immunofluorescence and cells were used in transduction efficiency, internalization and flow cytometry assays.

### Determination of CAR and integrin expression by flow cytometry.

Flow cytometry was used to determine the cell surface expression of CAR, αv integrin subunit, and integrin heterodimers αvβ3 and αvβ5. Adherent cells, cultured up to 80% of confluence, were detached by trypsin (Sigma-Aldrich, USA) and washed twice with PBS. For each sample 5 × 10^5^ cells were used. Subsequently, the cells were incubated on ice for 1 h with the specific unlabeled primary antibodies that recognized CAR (05-644 RmcB, Merck Milipore, Germany), αv integrin subunit (407286 272-17E6, Merck Milipore, Germany), integrin heterodimers αvβ3 (MAB1976 LM609, Merck Milipore, Germany) and αvβ5 (MAB1961 P1F6, Merck Milipore, Germany) and isotype control (M5284, Sigma-Aldrich, USA), while its binding was revealed by incubation on ice for 1 h with FITC-conjugated anti-mouse antibody (554001, BD Biosciences, USA) as a secondary reagent. Flow cytometry was performed on FACSCalibur (BD Biosciences, USA), while cell acquisition was made using BD CellQuest software package (BD Biosciences, USA). Data were analyzed using FCS Express 3 (*De Novo* Software, USA), and showed as transduction efficiency corresponding to the geometric MFI shown as an absolute or relative value.

### Isolation of A549 cells clones.

To construct A549-shCLTC and A549-shCAV-1 clones with decreased expression of clathrin or caveolin-1, A549 cells were transfected with plasmids containing antibiotic resistance genes and short hairpin RNA (shRNA) specific for a particular protein. To construct A549-shCLTC clones, pSUPER vector system for the expression of shRNA was used (OligoEngine, USA). Briefly, oligonucleotides were constructed according to the (48). Oligonucleotides P1: 5′-GATCCCCTGAGCTGTTTGAAGAAGCATTCAAGAGATGCTTCTTCAAACAGCTCATTTTTA-3′ and P2: 5′-AGCTTAAAAA TGAGCTGTTTGAAGAAGCATCTCTTGAATGCTTCTTCAAACAGCTCAGGG-3′ were annealed and cloned into HindIII (NEB, UK) and BglII (NEB, UK) digested vector. Resulting plasmid pSUPER-shCLTC and vector pSUPER were purified using Miniprep columns (Qiagen, Germany) and transfected into A549 cells using Lipofectamine (Thermo Fisher Scientific, USA) according to the manufacturer’s protocol. Clone A549-shCLTC-NEG is transfected with pSUPER plasmid, and clones A549-shCLTC-58 and -69 with pSUPER-shCLTC plasmid. Clones were selected in the presence of puromycin (1 μg/mL, Sigma-Aldrich, USA) and screened for clathrin expression measured by Western blotting analysis. To construct A549-shCAV-1 clones, A549 cells were transfected with pSuper-Neo plasmid containing shCAV1 described in (49) using Lipofectamine (Thermo Fisher Scientific, USA) according to the manufacturer’s protocol. Clones were selected in the presence of G418 (0.6 mg/mL, Sigma-Aldrich, USA) and screened for caveolin-1 expression measured by Western blotting analysis.

### Internalization by confocal microscopy.

Cells were seeded in 24-well plates on coverslips at a density of 2 × 10^4^ cells per well in antibiotic-free DMEM supplemented with 10% (vol/vol) FBS and 48 h later labeled viruses were added to the cells (HAdV26-AF488, 2 × 10^4^ vp/cell; HAdV5-AF488, 5 × 10^3^ vp/cell) for 30 min on ice to allow virus binding, and then placed at 37°C, 5% CO_2_ for 1 h to allow virus internalization. Noninternalized viruses were removed by washing the cells twice with PBS and cells were fixed with 2% paraformaldehyde in PBS for 12 min at room temperature. Next, cells were permeabilized (0.1% Triton X-100 in PBS for 2 min at room temperature), blocked with 3% BSA/PBS for 30 min at room temperature and incubated with the appropriate primary antibodies for 1 h at room temperature, followed by incubation with the appropriate secondary antibody for 1 h at room temperature. Cells were then incubated with Alexa Fluor 555 Phalloidin (Thermo Fischer Scientific, USA) for 15 min at room temperature. In experiments where the antibodies were not used, Alexa Fluor 555 Phalloidin was added immediately after permeabilization. Coverslips were mounted in Fluoromount G (Southern Biotech, USA) containing DAPI for nuclei staining. The following primary antibodies were used: anti-clathrin (CLTC, ab21679, Abcam, UK), anti-caveolin-1 (CAV-1, ab2910, Abcam, UK), anti-dynamin-2 (DNM2, ab3457, Abcam, UK). Leica TCS SP8 X inverted confocal microscope (Leica Microsystems, Germany) with 63 x/1.40 oil-immersion objective was used for imaging. The images were analyzed using LAS X (Leica Microsystems, Germany) and ImageJ (NIH, USA) software and they are showing maximum projections of confocal stacks, unless otherwise indicated. The qualification of HAdV internalization in cells is presented as the number of virus particles per cell, with mean and standard deviations. The N number represents the number of cells analyzed in each sample.

### Western blot analysis.

Expression of dynamin-2, caveolin-1 and clathrin after their downregulation by specific siRNA or in A549-shCLTC and A549-shCAV-1 clones was assessed by Western blotting analysis. Cells (3 × 10^5^ cells per sample) were lysed with Laemmli buffer heated to 95°C (150 μL/sample), scraped off the plate, sonicated and boiled (95°C, 3 min). Proteins were separated using 10% acrylamide gel by SDS-PAGE and transferred to nitrocellulose membrane (Amersham Protran, GE Healthcare, USA). The membrane was blocked with 5% (wt/vol) nonfat dry milk (Carl Roth, Germany) in Tris-Buffered Saline containing 1% Tween 20 and probed with the appropriate primary antibodies, followed by incubation appropriate horseradish peroxidase-conjugated IgG secondary antibody. Detection was performed with Pierce ECL Western Blotting Substrate (Thermo Fischer Scientific, USA), using ChemiDoc Imaging System (Bio-Rad, USA). Densitometry was performed with ImageJ software (NIH, USA). The following antibodies were used: anti CLTC (ab21679, Abcam, UK), anti CAV-1 (ab2910, Abcam, UK), anti DNM-2 (ab3457, Abcam, UK). Proteins were normalized to the total protein stained with amidoblack and the results were presented as relative expression of proteins compared to control cells.

### Use of chemical inhibitors of endocytosis.

Transduction efficiency, binding, and internalization of HAdV and αvβ3 integrin expression on the cell surface were also determined after the use of chemical inhibitors of endocytosis pitstop 2 and dyngo 4a. In all experiments using chemical inhibitors of endocytosis, the culture medium was antibiotic-free DMEM supplemented with 0.2% (vol/vol) FBS. Dyngo 4a (100 mM, ab120689, Abcam, UK) and pitstop 2 (30 mM, ab120687, Abcam, UK) stock solutions were prepared by dissolving lyophilized inhibitors in DMSO. The stock solutions were stored at −20°C. Dilutions were prepared in culture medium.

For the measurement of transduction efficiency in the presence of dyngo 4a or pitstop 2, cells were incubated with culture medium (control sample), dyngo 4a (25 μM) or pistop 2 (20 μM) in culture medium at 37°C, 5% CO_2_ for 30 min prior to incubation with viruses. The subsequent steps are the same as those described in the chapter transduction efficiency assay.

For binding cells were seeded in 6-well plates on coverslips at a density of 3 × 10^5^ cells per well in antibiotic-free DMEM supplemented with 10% (vol/vol) FBS, and 24 h later incubated in 500 μL of culture medium (control sample) or pitstop 2 (20 μM) at 37°C, 5% CO_2_ for 30 min, and then for 5 min on ice. HAdV were added to cells (10^3^ vp/cell) and incubated for 1 h on ice. The subsequent steps are the same as those described in the chapter adenovirus binding.

For the measurement of internalization in the presence of pitstop 2, cells were seeded in 24-well plates on coverslips at a density of 2 × 10^4^ cells per well in antibiotic-free DMEM supplemented with 10% (vol/vol) FBS and 48 h later incubated with culture medium (control sample) or pitstop 2 (20 μM) at 37°C, 5% CO_2_ for 30 min. Labeled viruses were added to the cells (HAdV26-AF488 2 × 10^4^ vp/cell) and incubated at 37°C, 5% CO_2_ for 1 h. After washing unbound viruses twice with PBS, the cells were fixed in the no recovery condition, while in recovery condition were fixed after additional incubation at 37°C, 5% CO_2_ for 1 h in antibiotic-free DMEM supplemented with 10% (vol/vol) FBS. The samples are fixed, permeabilized, incubated with Alexa Fluor 555 Phalloidin and mounted as described in the chapter internalization by confocal microscopy.

For the measurement of αvβ3 integrin expression on the cell surface in the presence of pitstop 2, cells were seeded in 6-well plates at a density of 3 × 10^5^ cells per well in antibiotic-free DMEM supplemented with 10% (vol/vol) FBS and 48 h later incubated with culture medium (control sample) or pitstop 2 (20 μM) at 37°C, 5% CO_2_ for 90 min. Cells were then washed twice with cold PBS and detached by cold trypsin followed by the same procedure as described in the chapter determination of CAR and integrin expression by flow cytometry.

### Statistical analysis.

All experiments were performed at least three times in duplicate or triplicate, except flow cytometry experiments, which were performed twice. All analyses and graphs were created in GraphPad Prism (GraphPad Software Inc., USA). Data were analyzed by unpaired Student's *t* test and expressed as mean ± standard deviation. ns, not significant; *, *P* < 0.05; **, *P* < 0.01; ***, *P* < 0.001, ****, *P* < 0.0001.
