# Supplementary material for: Human Adenovirus Type 26 Infection Mediated by αvβ3 Integrin Is Caveolin-1-Dependent
Source: Microbiol Spectr. 2022 Aug 4;10(4):e01097-22. doi: 10.1128/spectrum.01097-22 (PMC9430667; doi:10.1128/spectrum.01097-22)
Supplement: Supplemental file 1 — Supplemental material. Download spectrum.01097-22-s0001.pdf, PDF file, 1.1 MB [file spectrum.01097-22-s0001.pdf]

# Human adenovirus type 26 infection mediated by αvβ3 integrin is caveolin-1-dependent

Davor Nestić[1], Jerome Custers[2], Danijel Švec[1], Dragomira Majhen[1*]

[1] Division of Molecular Biology, Ruđer Bošković Institute, Zagreb, 10000, Croatia

[2] Viral Vaccine Discovery and Early Development, Janssen Vaccines and Prevention BV, Leiden, 2333CN, The Netherlands

* Corresponding author

E mail: dmajhen@irb.hr

## Supplemental material

## Supplemental figures

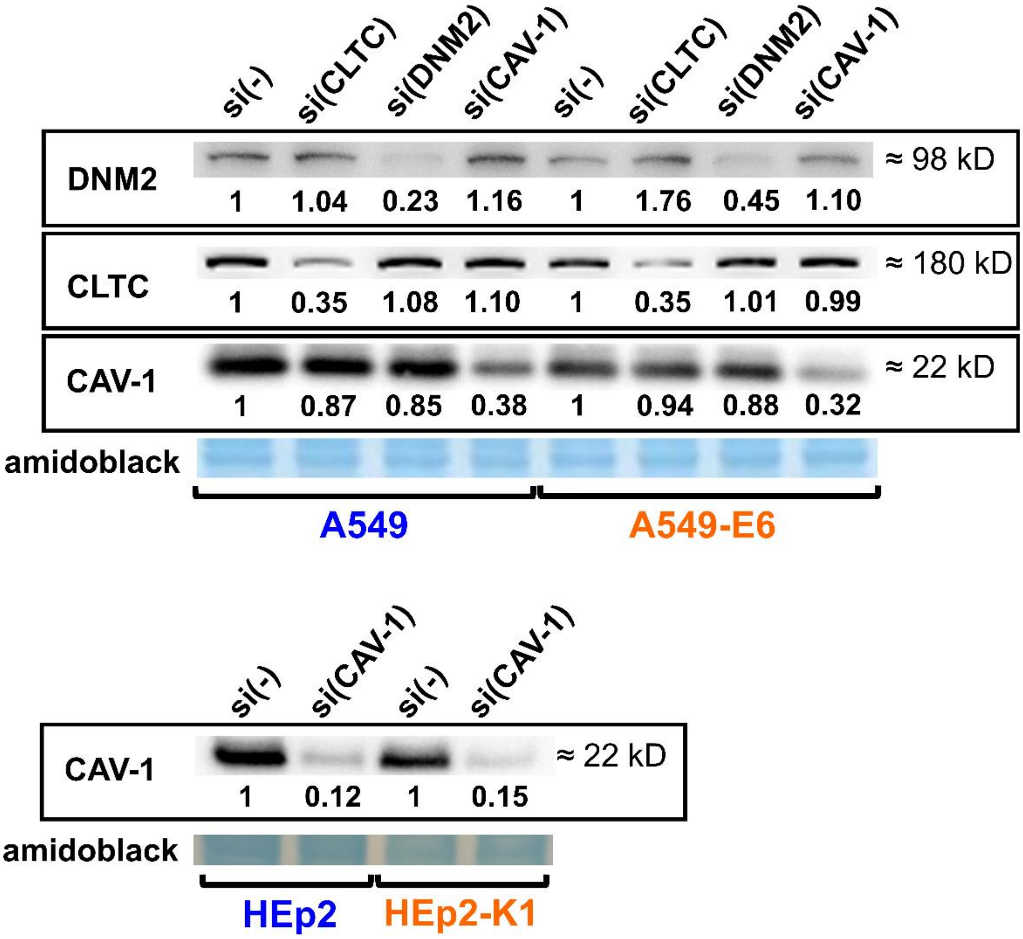


**Fig. S1. Expression of clathrin (CLTC), dynamin-2 (DNM2) and caveolin-1 (CAV-1) in studied cells.** Relative expression of CLTC, DNM2 and CAV-1 in A549 and A549-E6 cells and relative expression of CAV-1 in Hep2 and Hep2-K1 cells after downregulating with specific siRNA. The numbers below the bands show protein expression in a sample of particular cell line relative to the expression in si (-) that was set as 1.

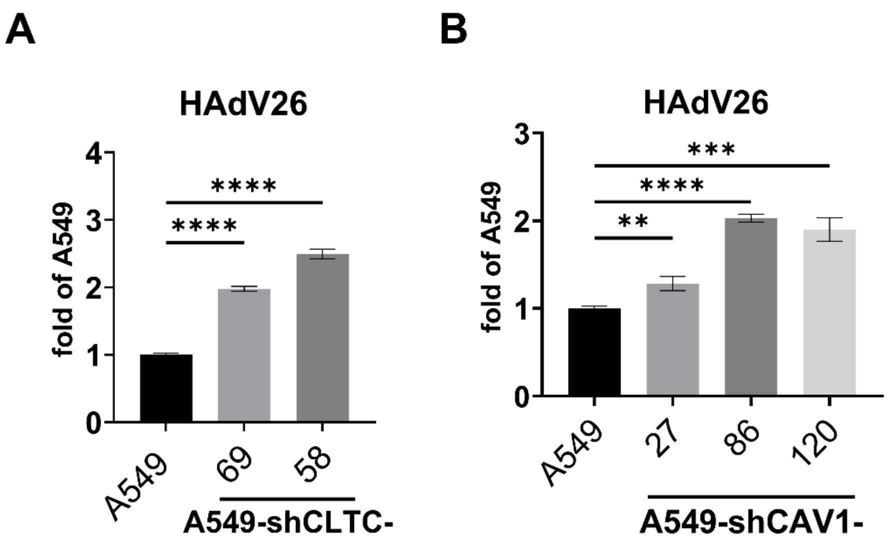


**Fig. S2. Binding of HAdV26 in A) A549-shCLTC clones with decreased expression of CLTC and B) A549-shCAV-1 clones with decreased expression of CAV-1.** Cells were incubated with HAdV26 on ice for 1 h, at multiplicity of infection (MOI), $10^3$ viral particles (vp)/cell. Total (cellular plus viral) DNA was extracted from the cells and used for quantification of viral DNA by qPCR, using the CMV region as a target sequence. Data are presented as representative data of two independent experiments in triplicates which yielded comparable results and shown as fold of value obtained for A549 cells ± standard deviation. **P < 0.01; ***P < 0.001; ****P < 0.000.1.

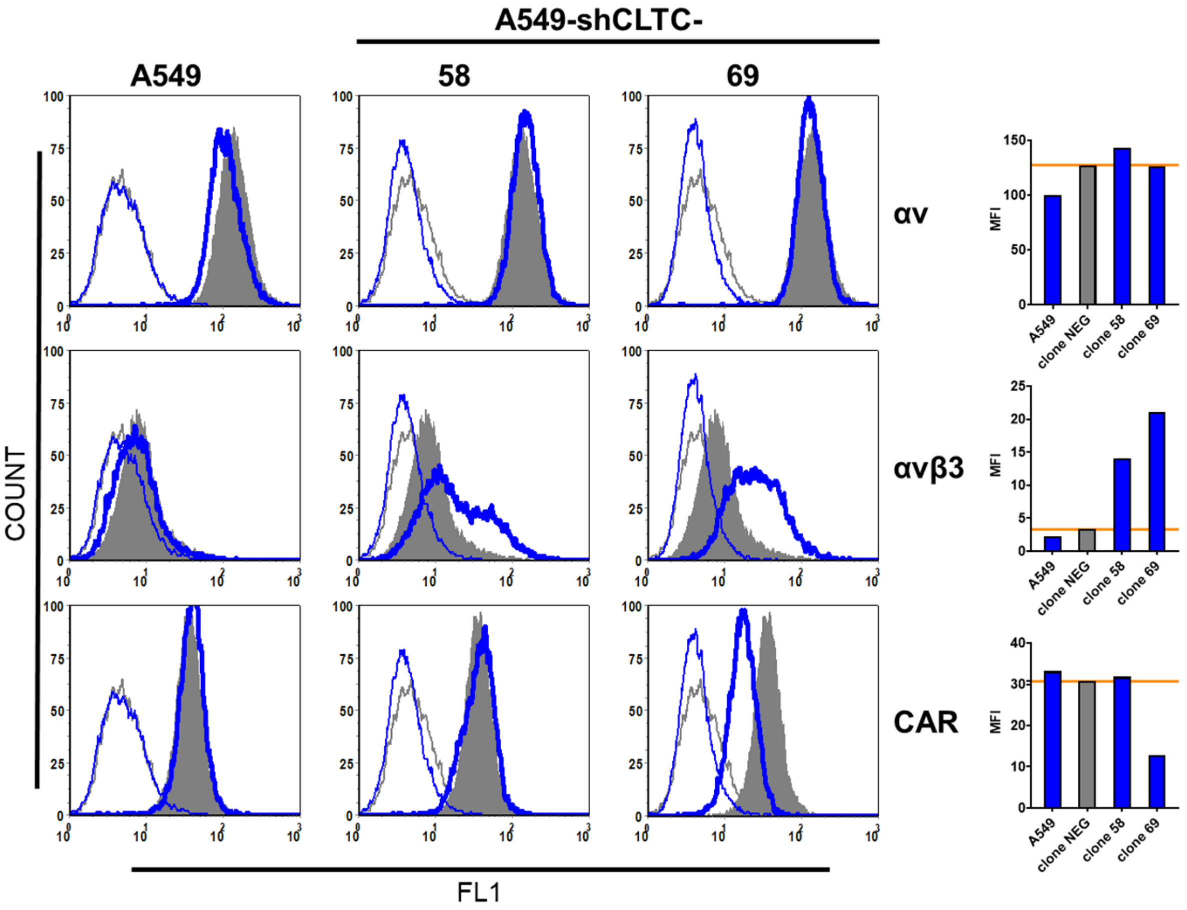


**Fig. S3. Expression of αv and αvβ3 integrins, and CAR on surface of A549 and A549-shCLTC-58 and -69 clones with decreased expression of CLTC in comparison to A549-shCLTC-NEG clone (gray).** Expression of αv and αvβ3 integrins, and CAR on surface of cells was determined by flow cytometry. Thin lines histograms represent isotype controls and bold lines or filled histograms the expression of the corresponding protein. Results from left panel were presented as comparisons of geometric mean fluorescence intensities (MFI) between cell lines (right panel). The representative data of two independent experiments which yielded comparable results are shown (n = 2).

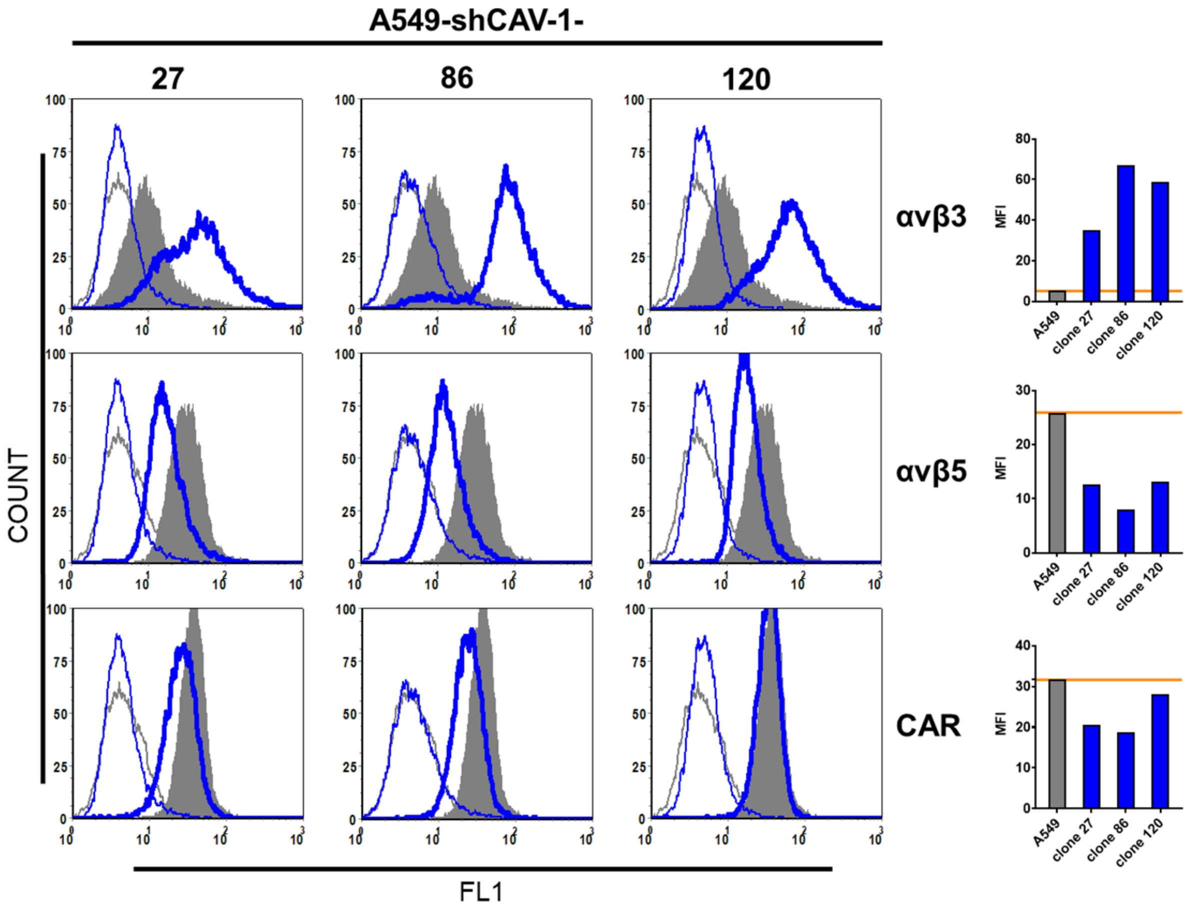


**Fig. S4. Expression of αvβ3 and αvβ5 integrins, and CAR on surface of A549-shCAV-1-27, -86 and -120 clones with decreased expression of CAV-1 in comparison to A549 cells (gray).** Expression of αvβ3 and αvβ5 integrins, and CAR on surface of cells was determined by flow cytometry. Thin lines histograms represent isotype controls and bold lines or filled histograms the expression of the corresponding protein. Results from left panel were presented as comparisons of geometric mean fluorescence intensities (MFI) between cell lines (right panel). The representative data of two independent experiments which yielded comparable results are shown (n = 2).

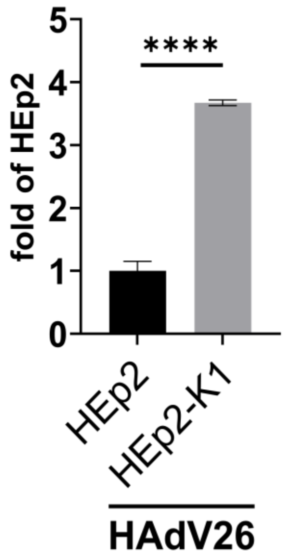


**Fig. S5. Transduction efficiency of HAdV26 in HEp2 and HEp2-K1 cells.** Transduction efficiency was measured by flow cytometry 48 h after infection. Data are presented as representative data of two independent experiments in triplicates which yielded comparable results and shown as fold of value obtained for HEp2 cells ± standard deviation. **P < 0.01; ***P < 0.001; ****P < 0.000.1.
